# Correlation between vitamin D3 serum levels and jaw bone density of candidates for dental implant treatment using CBCT

**DOI:** 10.34172/joddd.025.41648

**Published:** 2025-03-31

**Authors:** Ali Mesgarzadeh, Amir Zandesh, Hooman Sojoudi, Ali Hossein Mesgarzadeh, Farzad Esmaeili, Emran Hajmohammadi

**Affiliations:** ^1^Faculty of Dentistry, Tabriz University of Medical Sciences, Tabriz, Iran; ^2^Student of Dentistry, Faculty of Dentistry, Tabriz University of Medical Sciences, Tabriz, Iran; ^3^Department of Oral and Maxillofacial Surgery, Faculty of Dentistry, Tabriz University of Medical Sciences, Tabriz, Iran; ^4^Department of Oral and Maxillofacial Radiology, Faculty of Dentistry, Tabriz University of Medical Sciences, Tabriz, Iran; ^5^Department of Oral and Maxillofacial Surgery, Faculty of Dentistry, Ardabil University of Medical Sciences, Ardabil, Iran

**Keywords:** Bone density, CBCT, Dental implants, Vitamin D3

## Abstract

**Background.:**

Little information is available about the effect of vitamin D on jaw bone density, and human studies about this entity are scarce. Vitamin D deficiency weakens bone regeneration and is responsible for many systemic diseases, such as chronic kidney disease, diabetes mellitus, etc.

**Methods.:**

Fifty candidates for dental implant treatment aged 20‒60 were randomly chosen at the Implantology Department of the Faculty of Dentistry, Tabriz University of Medical Sciences. Fifteen patients were male, and 35 were female, and they were examined for their vitamin D3 serum levels. All the patients had cone-beam computed tomography (CBCT) radiographic images. According to their vitamin D3 levels, they were split into three groups: deficient, insufficient, and sufficient, and the density of bone was evaluated using the mean calculated Hounsfield unit from the Planmeca Romexis software.

**Results.:**

In the vitamin D3-deficient group, the mandibular bone of both males and females demonstrated lower bone densities; however, there was no significant correlation between bone density and vitamin D3 serum levels in either the maxilla or the mandible.

**Conclusion.:**

This study could not find a correlation between the serum levels of vitamin D3 and the bone density of the jaws. Further studies are necessary in this respect.

## Introduction

 Vitamin D has a distinctive role in human health, survival, and fertility. Several studies have emphasized its role in preventing various pathologic conditions, including cardiovascular diseases, malignant conditions, inflammatory bowel disease, multiple sclerosis, rheumatoid arthritis, type 1 diabetes, immune system disorders, and infectious processes. Additionally, calcium and phosphorus absorption is improved by vitamin D, the two most essential ions of bone, from the intestines, reducing their excretion from the kidneys and strengthening bone formation. Vitamin D deficiency is considered one of the critical factors in bone metabolism disorders.^1,2^

 Measuring bone density can serve as a predictive sign for fractures resulting from osteoporosis. However, vitamin D significantly impacts the metabolism of calcium and phosphorus. The amount of 25-hydroxyvitamin D in the blood is well recognized as an indicator of a person’s vitamin D status.^3,4^

 Wical and Brussee reported that patients taking vitamin D supplements experienced significantly less bone loss after getting immediate dentures compared to those who did not take supplements.^5^

 The development of dental implants to achieve osseointegration has become a widely used method for restoring oral function. Knowing the Hounsfield number as a method of bone density measurements for implant placement can help surgeons adopt appropriate treatment plans for patients with poor bone density.^6,7^

 Bazal-Bonelli et al,^8^ in their systematic review of the relationship between serum vitamin D levels and dental implants in terms of marginal bone loss, survival rates, and associated complications, found that cases with lower serum vitamin D levels were associated with slightly worse outcomes in terms of marginal bone loss.

 Recent research on the alveolar ridge and healing of freshly extracted tooth areas indicates that calcium and cholecalciferol (vitamin D3) supplements have systemic effects that accelerate bone regeneration. The quantity and quality of bone available at the implant placement site strongly affects the success rate of dental implant treatment. Studies show that in cases where bone quantity and quality are inadequate, the failure rate of implant treatment rises.^9,10^ Due to the rising demand for dental implants, their dependence on healthy bone structure, and the impact of vitamin D3 deficiency on bone, this study aims to measure serum levels of vitamin D and bone density in referring patients to the Dental Implant Department of Tabriz Faculty of Dentistry.

## Methods

 This cross-sectional study included 50 patients visiting the Dental Implant Department of the Tabriz Faculty of Dentistry.

 According to different sources,^11^ vitamin D deficiency was defined as having a vitamin D serum level < 20 ng/mL, while serum levels < 12 ng/mL are classified as severe deficiency.

###  Inclusion criteria

 All patients who were candidates for implant treatment in the posterior alveolar region, required cone-beam computed tomography (CBCT) imaging, and were willing to take part completed a written informed consent form at the start of the study.

###  Exclusion criteria

 Patients who did not want a serum level of vitamin D3 test, patients suffering from a systemic disease related to bone metabolism, patients taking vitamin D3 supplements, and post-menopause women were excluded.

 Patients who were candidates for implantation in the posterior alveolar areas and had CBCT images were selected randomly. They were ordered a free serum vitamin D3 test. The test was conducted in a private facility using a standard kit and measured by one person.

 A trained radiologist implemented the Hounsfield number to quantify the bone density in the posterior edentulous zones extending from the first premolar to the wisdom teeth. All the patients were examined using a CBCT machine (Newtom VGi) at Tabriz Faculty of Dentistry.

 Bone density was measured about one millimeter away from the inferior alveolar canal and the maxillary sinuses to avoid these structures. Only the trabecular bone was included in the measurements, and the cortical bone was excluded to ensure consistent results.

**Figure 1:**
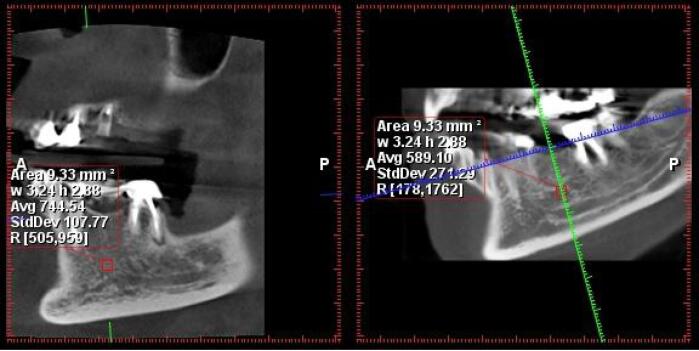
The measurement of bone density in the posterior mandibular region

**Figure 2:**
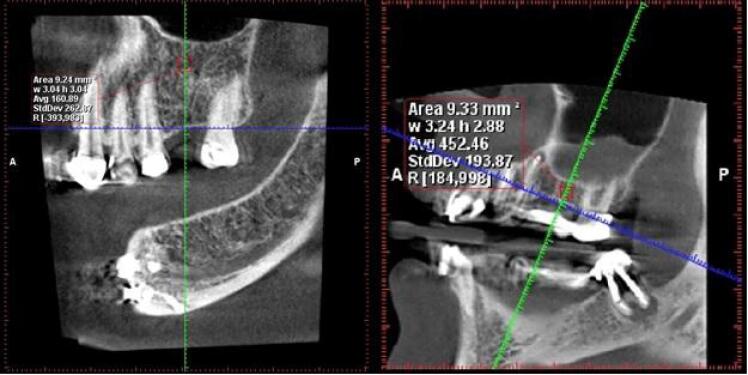
The measurement of bone density in the posterior maxilla region

 A specific measurement protocol was employed in the Romexis Planmeca software. The defined measurement area was a uniform mass of 3 × 3 mm. Within this area, the software automatically calculated the average Hounsfield numbers (as a measure of radiodensity) and presented them as an average value (Figures 1 and 2).

 This average value was typically obtained from the edentulous areas corresponding to tooth numbers #6 and #5. If those areas were not available, measurements were taken from other posterior regions. Notably, the measurements were made at a level with a 1-mm distance from both the inferior alveolar canal and the maxillary sinus floor.

 The findings were presented as means ± standard deviations (SD) and percentages. The Kruskal-Wallis test was used for comparison, and the Kolmogorov-Smirnov test was used to determine whether the data was normal. SPSS 24 was used for statistical analyses.

 A result was considered statistically significant if the *P* value was < 0.05.

## Results

 This study investigated the effect of vitamin D serum level on the density of jaw bones in 50 patients.

 In this study, 30% of patients were male and 70% were female (Table 1). The findings from Table 2 indicate that the mean vitamin D3 serum level in the studied patients was 30.12 ± 11.95 ng/mL, with a range of 4‒65.2 ng/mL in serum. Additionally, Table 3 shows that 22% of the patients had vitamin D3 deficiency, 18% had vitamin D3 insufficiency, and 60% had normal vitamin D3 levels. Bone density analysis showed an average value of 372.37 ± 143.88 in the maxilla and 436.21 ± 184.34 in the mandible (Table 4).

**Table 1:** The frequencies of examined patients by gender

	**Number**	**Percent**
Men	15	30.0 %
Women	35	70.0 %
Total	50	100.0 %

**Table 2:** Means and standard deviations and minimum and maximum amounts of vitamin D3 in the examined patients

	**Number**	**Minimum**	**Maximum**	**Mean**	**SD**
Vitamin D3	50	4.00	65.20	30.12	11.95

SD: standard deviation.

**Table 3:** A comparison of patient frequencies according to body levels of vitamin D3

	**Number**	**Percent**
Deficiency	11	22.0
Insufficient	9	18.0
Normal	30	60.0
Total	50	100.0

Deficiency: < 20 ng/mL vitamin D3 serum level. Insufficient: 20‒30 ng/mL vitamin D3 serum level. Normal: > 30 ng/mL vitamin D3 serum level.

**Table 4:** Means and standard deviations and minimum and maximum values of bone density in the maxilla and mandible in the examined patients

	**Number**	**Minimum**	**Maximum**	**Mean**	**SD**
Maxilla	39	144.00	733.50	372.37	143.88
Mandible	38	115.50	800.50	436.21	184.34

SD: standard deviation.

 This study revealed no significant difference in bone density between the maxilla and mandible in male patients across various serum levels of vitamin D3. There was a slight increase in bone density in the mandible with higher vitamin D3 levels, but it was not statistically significant. Similarly, in female patients, across different vitamin D3 levels, there was no significant difference in the maxilla and mandible’s bone density. However, women with vitamin D3 deficiency had the lowest bone density in both the maxilla and mandible, though this finding was not statistically significant (Tables 5 and 6).

**Table 5:** Comparison of bone density based on vitamin D3 group in both jaws and in men and women (*P* value: Kruskal-Wallis)

	**Vitamin D3**	**Men**			**Women**		
		**Number**	**Mean**	**SD**	**Number**	**Mean**	**SD**
Maxilla	Deficiency	4	470.50	248.71	4	270.13	142.38
	Insufficient	3	399.67	167.69	4	462.13	96.97
	Normal	3	446.17	27.59	20	351.50	117.49
	Total	10	441.95	167.34	28	355.68	125.65
	*P* value	0.885			0.089		
Mandible	Deficiency	5	464.70	87.96	5	216.60	108.11
	Insufficient	4	576.00	168.68	2	443.25	22.27
	Normal	3	630.50	119.62	19	425.66	186.53
	Total	12	543.25	135.81	26	386.81	184.76
	*P* value	0.220			0.066		

SD: standard deviation. Deficiency: < 20 ng/mL vitamin D3 serum level. Insufficient: 30-20 ng/mL vitamin D3 serum level. Normal: > 30 ng/mL vitamin D3 serum level.

**Table 6:** Comparison of bone density based on vitamin D3 group in two jaws (*P* value: Kruskal-Wallis)

**Vitamin D3**	**Maxilla**			**Mandible**		
	**Number**	**Mean**	**SD**	**Number**	**Mean**	**SD**
Deficiency	8	370.31	216.03	10	340.65	160.41
Insufficient	7	435.36	123.25	6	531.75	147.88
Normal	23	363.85	114.26	22	453.59	190.69
Total	38	378.38	140.76	38	436.21	184.34
*P* value	0.505			0.104		

SD: standard deviation. Deficiency: < 20 ng/mL vitamin D3 serum level. Insufficient: 30-20 ng/mL vitamin D3 serum level. Normal: > 30 ng/mL vitamin D3 serum level.

## Discussion

 The quality and quantity of bone influence dental implant treatment success in the implant placement area. Previous studies have shown that implant failure rates increase in bones with poor quantity and quality.^7^

 In the present study, 50 patients underwent implant surgery; 30% of patients were male, and 70% were female. 22% of patients had vitamin D3 deficiency, 18% were in the insufficient range, and 60% were in the normal range. Bone density in both the maxilla and mandible showed no significant relationship with serum vitamin D3 levels. Also, there was no significant difference in the average bone density in different vitamin D3 groups.

 Werny et al, in a systematic review of animal and human studies, indicated that lower vitamin D serum levels negatively affect implant osseointegration in animals. In animals with systemic disorders such as osteoporosis, diabetes mellitus, chronic renal disease, and vitamin D deficiency, vitamin D supplements improve osseointegration. There is some evidence to support the theory that vitamin D improves osseointegration in humans in a similar way. The results of this study contradict the present study.^12^

 Acipinar et al,^13^ in a study of 90 dental implant sites, evaluated the peri-implant sulcus fluid 25-hydroxy-vitamin D3 (25(OH)D3) levels in peri-implant healthy tissues and peri-implant diseases and indicated that the 25(OH)D3 concentration was significantly lower in the peri-implantitis group. Similarly, Singh et al^14^ found a positive correlation between vitamin D serum levels and crestal bone loss on CBCT in their investigation of the relationship between serum vitamin D and crestal bone level in dental implant patients using CBCT.

 In a retrospective study in 2016, Mangano et al^15^ investigated the association of vitamin D serum level with early implant failure in 1625 implants. Although that study reported an increasing trend in the incidence of primary implant failure with vitamin D deficiency, there was no significant difference in the frequency of primary implant failure across the three vitamin D groups studied, consistent with the present investigation.

 Examining the serum level of vitamin D3 in patients depends on factors such as age, gender, body mass index, exposure to sunlight, skin pigmentation severity, and type of nutrition.^16-18^ These factors can act as confounding variables in the current study and similar studies. However, in the present study, some controllable factors, such as the patients consuming vitamin D3 supplements and women after menopause, were excluded.

 Research has shown that vitamin D stimulates the activity of bone cells, and in patients who have received enough vitamin D, bone density is higher, and bone fractures are fewer.^19^ Conversely, vitamin D is essential in regulating the dynamic state of bone and controlling the activity of bone cells like osteoblasts and osteoclasts. Researchers have shown that vitamin D has an anabolic effect on osteoblasts.^20^ Consequently, a recent trend in the field of dental implant success is to promote immune system regulation to achieve quicker and more effective osseointegration.^21,22^ Therefore, vitamin D stimulates osteoblasts to synthesize several factors that promote osseointegration and bone formation.^23^

 Schulze-Späte et al^24^ showed in a 2016 clinical trial that vitamin D3 plus calcium raises serum vitamin D levels and affects bone regeneration to improve maxillary sinus augmentation. Nevertheless, there were no statistically significant differences in graft resorption or bone growth between the supplement therapy and control groups.

 In a 2015 study on the regenerative response of alveolar bone to topical calcitriol application in vitamin D-deficient rats, Fügl et al^25^ demonstrated that topical calcitriol application does not promote bone healing and that vitamin D deficiency does not always have a negative effect on bone regeneration in the rat jaw.

 In a review of animals, Javed et al^26^ showed five studies that indicated the significant effect of vitamin D3 supplementation on increasing new bone formation or bone-implant contact around the implants.

 In the present study, bone density was evaluated using CBCT images. CBCT images use a reduced radiation dose and are one way to evaluate bone quality before placing an implant.

 Shapurian et al^6^ showed that Hounsfield values, as a quantitative indicator of bone density, could be a useful diagnostic tool, provide an objective assessment of bone density for the implant surgeon, and lead to the modification of surgical procedures, especially in suspected conditions of low bone quality.

 In the 2014 study by Razi et al,^27^ the relationship between grayscale in CBCT and HU in CT was examined. It showed that the grayscale in CBCT is a criterion for assessing bone density before implant treatment, and due to low radiation and the low cost of CBCT, it is the method of choice compared to CT scan.

 According to the current study, clinical studies with higher sample sizes are recommended to reach a definite conclusion about the relationship between the serum levels of vitamin D and bone density.

## Conclusion

 This study found no significant relationship between serum vitamin D3 levels and bone density in the maxillary and mandibular regions. The bone density of men and women in the various vitamin D3 groups did not differ significantly. However, in the mandible, the lowest bone density was observed in patients with vitamin D3 deficiency.

## Competing Interests

 The authors deny any conflict of interests related to this study.

## Ethical Approval

 The present study was approved by the Ethics Committee of Tabriz University of Medical Sciences under the code IR.TBZMED.REC.1398.1267.
